# Unexpected gaps in knowledge of familial hypercholesterolaemia among Dutch general practitioners

**DOI:** 10.1007/s12471-024-01862-y

**Published:** 2024-04-04

**Authors:** Shirin Ibrahim, Jim N. de Goeij, Nick S. Nurmohamed, Jing Pang, Sibbeliene E. van den Bosch, Fabrice M. A. C. Martens, Jeanine E. Roeters van Lennep, Willemijn Corpeleijn, Talip Tumkaya, G. Kees Hovingh, Gerald F. Watts, Erik S. G. Stroes, Laurens F. Reeskamp

**Affiliations:** 1 Department of Vascular Medicine, Amsterdam University Medical Centres, location Amsterdam University Medical Centre-University of Amsterdam, Amsterdam, The Netherlands; 2 Department of Cardiology, Amsterdam University Medical Centres, location Vrije Universiteit medical centre, Amsterdam, The Netherlands; 3 Medical School, University of Western Australia, Perth, WA Australia; 4 Department of Paediatrics, Division of Metabolic Disorders, Emma Children’s Hospital, Amsterdam University Medical Centres and Gastroenterology, Endocrinology & Metabolism (AGEM), location Academic Medical Centre-University of Amsterdam, Amsterdam, The Netherlands; 5 Department of Internal Medicine, Cardiovascular Institute, Erasmus Medical Centre, Rotterdam, The Netherlands; 6 Department of General Practice, Huisartsenpraktijk Parkhof, Maassluis, The Netherlands; 7 Department of Cardiology, Royal Perth Hospital, Perth, WA Australia

**Keywords:** Familial hypercholesterolaemia, Primary care, General practitioners, Questionnaire

## Abstract

**Background:**

Familial hypercholesterolaemia (FH) warrants early diagnosis to prevent premature atherosclerotic cardiovascular disease (CVD). However, underdiagnosis and undertreatment of FH persist. This study aimed to assess the knowledge and practice of FH care among general practitioners (GPs) in the Netherlands.

**Methods:**

An internationally standardised, online questionnaire was sent to Dutch GPs between February 2021 and July 2022. The survey assessed knowledge and awareness of FH, encompassing general familiarity, awareness of management guidelines, inheritance, prevalence, CVD risk, and clinical practice related to FH. Comparative analysis was performed using data on primary care physicians from Western Australia, the Asia-Pacific region and the United Kingdom.

**Results:**

Of the 221 participating GPs, 62.4% rated their familiarity with FH as above average (score > 4 on a 1–7 scale), with 91.4% considering themselves familiar with FH treatment and referral guidelines. Correct identification of the FH definition, typical lipid profile, inheritance pattern, prevalence and CVD risk was reported by 83.7%, 87.8%, 55.7%, 19.5%, and 13.6% of the respondents, respectively. Of the participants, 58.4% answered fewer than half of the 8 knowledge questions correctly. Dutch GPs reported greater FH familiarity and guideline awareness compared with their international counterparts but exhibited similar low performance on FH knowledge questions.

**Conclusion:**

Despite the Netherlands’ relatively high FH detection rate, substantial knowledge gaps regarding FH persist among Dutch GPs, mirroring global trends. Enhanced FH education and awareness in primary care are imperative to improve FH detection and ensure adequate treatment. Targeting the global suboptimal understanding of FH might require international efforts.

**Supplementary Information:**

The online version of this article (10.1007/s12471-024-01862-y) contains supplementary material, which is available to authorized users.

## Introduction

Familial hypercholesterolaemia (FH) is a highly prevalent genetic disorder specifically characterised by the life-long elevation of low-density lipoprotein (LDL) cholesterol plasma levels, which predisposes affected individuals to a 3- to 4‑fold increased risk of premature cardiovascular disease (CVD) [1–3]. Despite the well-established association with CVD, FH remains profoundly underdiagnosed and untreated worldwide [1, 4]. Estimates reveal that > 90% of the 30 million individuals affected by FH worldwide remain undiagnosed and only a minority of FH patients receive adequate treatment, while up to one-third receive no treatment at all [4, 5]. As early diagnosis and initiation of lipid-lowering therapy are key for therapeutic success in FH [6, 7], identification of FH index cases should preferably take place in an out-of-hospital setting due to the infrequent encounter of young patients in a hospital setting.

General practitioners (GPs) are particularly well-positioned for early identification of FH, as they are at the forefront of patient care, can initiate cascade screening and can more easily supervise the required lifelong lipid-lowering management at lower costs compared with other specialists [8]. Indeed, international guidelines have recommended that management and care for FH patients be primarily performed in a primary care setting [9]. This assertion is supported by the demonstrated success of various FH identification strategies in primary care settings. These include child-parent reverse cascade screening in primary care practices in the United Kingdom (UK) [10], as well as the use of electronic data extraction tools that employ algorithms to phenotypically diagnose FH [11, 12]. Use of such sophisticated data extraction tools not only increases the detection of FH in general practice but also helps to minimise practice workloads [8].

Knowledge and awareness of FH are pivotal factors for the successful detection and treatment of FH in primary care. However, survey studies conducted in the United States (US), UK, Western Australia, and the Asia-Pacific region reveal significant gaps in primary care physicians’ understanding of FH guidelines [13–16]. This includes limited awareness of the FH prevalence and heritability and the associated CVD risk, which are all crucial for assessing the pre-test probability of FH in individuals.

**Fig. 1:**
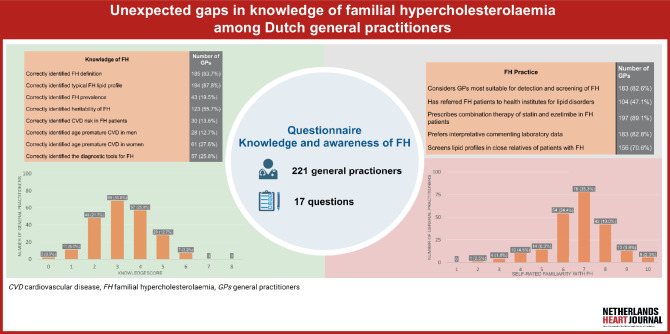
Infographic: Unexpected gaps in knowledge of familial hypercholesterolemia among Dutch general practitioners

The Netherlands has gained recognition as a leader in FH detection, owing to the implementation of a comprehensive nationwide cascade screening programme that ran from 1994 to 2013, which was demonstrated to be highly feasible, efficient, and effective in FH identification [17]. This initiative successfully identified 28,000 newly diagnosed FH patients. Consequently, it is hypothesised that the knowledge, awareness and practice of Dutch GPs regarding FH screening and treatment would surpass those of their international colleagues. The present study aimed to examine this hypothesis and assess gaps in knowledge, awareness, and clinical practice concerning the management of FH among GPs in the Netherlands, using a structured questionnaire (Fig. 1; [13]).

## Methods

### Study design and participants

GPs affiliated with the National Co-operation of Cardiology Centres (*Werkgroep Cardiologische centra Nederland, WCN*) in the Netherlands received an invitation via email from the WCN between February 2021 and July 2022, requesting participation in an anonymous questionnaire study on FH. To acquire a sample accurately representing different regions across the Netherlands, additional GP practices in regions underrepresented among the initial respondents of the survey were approached randomly. GPs were strictly instructed to answer the questions without any help or use of online sources.

Participants who had incompletely filled out questionnaires or submitted duplicates were excluded from the analysis. In addition, respondents not currently practicing as GPs were excluded. Upon reasonable request, the data that support the findings of this study are available from the corresponding author.

### Survey details

The study participants were presented an online survey based on a standardised questionnaire, which was previously developed and used in the ‘Ten Countries Study’ [13]. The ‘Ten Countries Study’ is a multinational research initiative that involved the participation of > 1000 primary care physicians, who completed a survey aimed at assessing their knowledge of FH and identifying gaps in their management of the disorder. The study enrolled primary care physicians from 10 countries and regions in the Asia-Pacific, including Australia, China, Hong Kong, Japan, Malaysia, Philippines, South Korea, Taiwan, and Vietnam, using the UK as the reference group. The survey was translated into Dutch and underwent several modifications and adaptations tailored to the current study (see Table S1 in Electronic Supplementary Material).

A total of 17 questions pertaining to FH were included in the adapted questionnaire, which were categorised into 3 sections: (1) awareness, (2) knowledge, and (3) preference, practice, and screening. The awareness-related questions encompassed various aspects, such as familiarity with the disorder itself and FH management guidelines. The level of familiarity with FH was evaluated by having the GPs self-rate their familiarity using a scale ranging from 1 to 7. This self-rating scale followed the approach outlined in the ‘Ten Countries Study’, where a score > 4 denoted above-average familiarity. To evaluate the GPs’ knowledge of FH, 8 questions covered areas such as the FH definition, hereditary pattern, prevalence and CVD risk. Lastly, the survey included 6 questions to assess GPs’ practices concerning FH.

In light of evolving guidelines after the development of the questionnaire, treatment-related questions were excluded from the survey. Furthermore, given the contemporary estimate of FH prevalence of 1:250, as opposed to the previous rough estimate of 1:500, this aspect was modified in the current study. Detailed information on the full questionnaire and modifications of the questionnaire used in the ‘Ten Countries Study’ can be found in Table S1 in the Electronic Supplementary Material.

### Statistical analysis

A ‘knowledge score’ was calculated for each participant based on their responses to the 8 knowledge-related questions. Each correctly answered question was assigned 1 point, resulting in a score ranging from 0 to 8. Survey responses between different demographic groups were compared using the chi-square test. The categorisation of these groups was based on a below-median versus above-median division. Additionally, survey responses from the participants in the present study were compared with those from countries included in the ‘Ten Countries Study’. Response frequencies were compared using a two-tailed binomial test and are expressed as odds ratio (OR), with the UK as reference group. Normally distributed data are reported as mean ± standard deviation (SD) and non-normally distributed data as median with interquartile range (IQR). All analyses were performed in R version 4.0.3. A two-sided *p* value < 0.05 was considered statistically significant.

## Results

### Demographic characteristics

A total of 246 respondents successfully completed the questionnaire, 25 of whom were excluded due to their lack of current engagement as practicing GP. The final analysis therefore comprised 221 GPs, whose demographic characteristics are presented in Tab. 1. Among the respondents, 122 (55.2%) were practicing in rural areas, while the remaining 99 (44.8%) practiced in metropolitan areas. In terms of practice setting, 105 GPs (47.5%) were affiliated with a group practice, while 75 (33.9%) worked in a duo practice and 41 (18.6%) in a solo practice. The median years of experience as a GP was 15.0 (IQR: 7.0–22.0), and the median number of diagnosed FH patients in the practice was 5.0 (IQR: 3.0–10.0).

**Table 1:** Demographic characteristics of Dutch general practitioners participating in online survey

Characteristic	Participants (*n* = 221)
Experience in general practice, years	15.0 (7.0–22.0)
Monthly patient load	334 ± 153
FH patients in practice	5.0 (3.0–10.0)
*Practice setting*	
Group	105 (47.5)
Duo	75 (33.9)
Solo	41 (18.6)
*Practice location*	
Rural	122 (55.2)
Metropolitan	99 (44.8)

Data are median (IQR), mean ± standard deviation or *n* (%)

*FH* familial hypercholesterolaemia

### Awareness

The survey responses are shown in Tab. 2. In the present study, 138 GPs (62.4%) rated their familiarity as above average (i.e. score > 4/7). Moreover, 202 (91.4%) reported being familiar with the FH treatment and referral guidelines and 122 GPs (55.2%) answered they were familiar with specialised health institutes for lipid disorders.

**Table 2:** Summary of survey findings on Dutch general practitioners’ awareness, knowledge, and practice regarding FH

Question	Participants (*n* = 221)
*Awareness*	
FH familiarity rated above average	138 (62.4)
Aware of FH guidelines	202 (91.4)
Aware of lipid disorder health institutes	122 (55.2)
*Knowledge*	
Correctly defined FH	185 (83.7)
Correctly identified FH lipid profile	194 (87.8)
Correctly identified FH prevalence	43 (19.5)
Correctly identified FH transmission rate to first-degree relatives	123 (55.7)
Correctly identified genetic testing is not required for FH diagnosis	57 (25.8)
Correctly identified CVD risk in untreated FH patients	30 (13.6)
Correctly identified age of premature CVD in men	28 (12.7)
Correctly identified age of premature CVD in women	61 (27.6)
*Practice*	
Assesses family history of CAD in patients with premature CAD	194 (87.8)
Screens lipid profile in close relatives of patients with FH	156 (70.6)
Performs screening for hypercholesterolaemia in children of families with premature CAD for age group:	
– 0–6 years	3 (1.4)
– 7–12 years	42 (19.0)
– 13–18 years	116 (52.5)
– > 18 years	34 (15.4)
– Unknown	26 (11.8)
Has referred FH patients to health institutes for lipid disorders (when aware of them)	104 (47.1)
*Preference*	
Considers general practitioners as suitable for early detection of FH and screening of first-degree relatives	183 (82.8)
Prefers alarming comment to be added to lipid profiles at risk for FH to enhance FH detection	183 (82.8)

Values are *n* (%)

*FH* familial hypercholesterolaemia, *CVD* cardiovascular disease, *CAD* coronary artery disease

GPs with ≥ 15 years of professional experience rated their familiarity with FH significantly more often as ‘above average’ compared with those with < 15 years of experience (OR 1.91; 95% CI: 1.11–3.31; *p* = 0.02) and were more familiar with specialised lipid institutes (OR: 2.86; 95% CI: 1.66–4.96; *p* < 0.001). Moreover, GPs with ≥ 5 FH patients in their practices also rated their familiarity with FH significantly more often as ‘above average’ compared with those with < 5 FH patients (OR: 2.17; 95% CI: 1.17–4.10; *p* = 0.01).

### Knowledge

General knowledge of FH among GPs was evaluated using 8 questions incorporated in the survey (Tab. 2, and see Table S1 in Electronic Supplementary Material). Of the GPs, 185 (83.7%) correctly identified the FH definition, while 194 (87.8%) correctly recognised the characteristic FH lipid profile. The correct prevalence of FH, its inheritance pattern, and the risk of atherosclerotic CVD (ASCVD) associated with FH were identified by 43 (19.5%), 123 (55.7%) and 30 GPs (13.6%), respectively. Of the participants, 118 (53.4%) underestimated the actual ASCVD risk associated with FH. The correct age for premature ASCVD in men and women was identified by 28 (12.7%) and 61 GPs (27.6%), respectively. The mean age given for premature ASCVD in women was 56.2 ± 8.8 years and 52.0 ± 8.9 years in men. Of the GPs, 80 (36.2%) perceived ASCVD as occurring prematurely in women at an age younger than that specified in national guidelines (< 60 years), while 45% regarded ASCVD as premature in men at a younger age than that outlined in the national guidelines (< 55 years).

Based on these items, a ‘knowledge score’ was constructed for each participant. Of the GPs, 129 (58.4%) answered < 4 of the 8 questions correctly and none answered > 6 questions correctly (see Figure S1 in Electronic Supplementary Material). GPs with ≥ 15 years of professional experience demonstrated mean knowledge scores ≥ 4 significantly more often compared with those with < 15 years of experience (OR: 2.03; 95% CI: 1.17–3.54; *p* = 0.01).

### Management

Of the respondents, 183 (82.8%) regarded their own profession as the most suitable for early detection and cascade screening of FH and only 105 (47.4%) referred their FH patients to a specialised health institute for lipid disorders. Regarding FH management, 156 respondents (70.6%) screened lipid profiles in first-degree relatives of FH patients and 194 (87.8%) screened patients with premature coronary artery disease (CAD) for a family history of CAD. Furthermore, 114 GPs (51.8%) initiated lipid profile screening in adolescents aged 13–18 years in families with premature CAD. FH screening in children before the age of 13 was undertaken by 45 (20.4%) of the participating GPs. Implementation of interpretative commenting on laboratory lipid profiles, which alerts physicians to the potential presence of FH, was endorsed by 183 GPs (82.8%), thereby acknowledging its potential to enhance identification of FH in primary care settings.

### International comparison

**Fig. 2:**
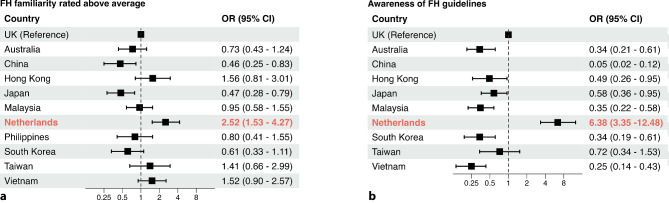
Comparison of primary care physicians’ responses to awareness-related questions between different countries/regions. **a** Self-rated familiarity with familial hypercholesterolaemia (*FH*) above average and **b** awareness of FH guidelines. Odds ratios (ORs) with 95% confidence interval (CI) derived from Pang et al. [13] are shown, with addition of OR with 95% CI for the Netherlands; United Kingdom (*UK*) is reference country

**Fig. 3:**
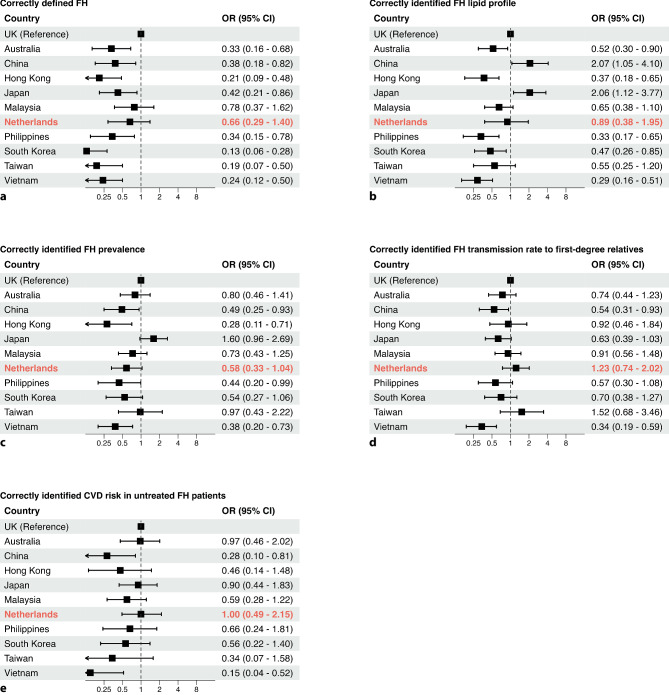
Comparison of primary care physicians’ responses to knowledge-related questions between different countries/regions. Correct identification of **a** familial hypercholesterolaemia (*FH*) definition, **b** typical FH lipid profile, **c** FH prevalence, **d** FH hereditary pattern and **e** cardiovascular disease (*CVD*) risk associated with FH. Odds ratios (ORs) with 95% confidence interval (CI) derived from Pang et al. [13] are shown, with addition of OR with 95% CI for the Netherlands; United Kingdom (*UK*) is reference country

**Fig. 4:**
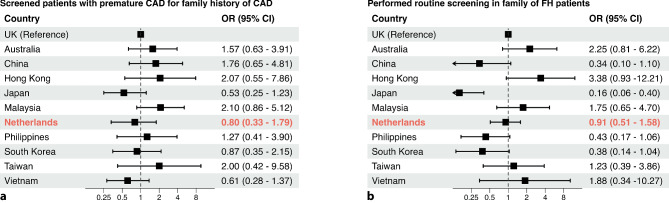
Comparison of primary care physicians’ responses to practice-related questions between different countries/regions. **a** Performing screening of patients with premature coronary artery disease (*CAD*) for family history of CAD and **b** performing routine screening of family members of patients with familial hypercholesterolaemia (*FH*). Odds ratios (ORs) with 95% confidence interval (CI) derived from Pang et al. [13] are shown, with addition of OR with 95% CI for the Netherlands; United Kingdom (*UK*) is reference country

Dutch GPs rated their familiarity with FH higher compared with primary care physicians from the UK (OR: 2.52; 95% CI: 1.53–4.27) (Fig. 2a). Among the countries participating in the ‘Ten Countries Study’, the percentage of self-perceived familiarity with FH rated as above average was < 50% [13], whereas it was 62.4% in the Netherlands. A similar difference was observed for self-perceived awareness of FH guidelines (OR: 6.38; 95% CI: 3.35–12.48) (Fig. 2b). With respect to the FH-related knowledge questions pertaining to the definition, typical lipid profile, prevalence, hereditary pattern, and associated CVD risk, Dutch GPs exhibited comparable scores as the primary care physicians in the UK (OR: 0.66; 95% CI: 0.29–1.40; OR: 0.89; 95% CI: 0.38–1.95; OR: 0.58; 95% CI: 0.33–1.04; OR: 1.23; 95% CI: 0.74–2.02; and OR: 1.00; 95% CI: 0.49–2.15, respectively) (Fig. 3). Comparable proportions of GPs conducted screenings for family history of CAD in patients with premature CAD in the ‘Ten Countries Study’ and the Netherlands (Fig. 4a). Moreover, Dutch GPs performed routine family screening in FH patients at a frequency comparable to that of primary care physicians in the UK (OR: 0.91; 95% CI: 0.51–1.58) (Fig. 4b).

## Discussion

The current study is the first to elucidate significant knowledge gaps in FH care among Dutch GPs, despite their self-perceived high familiarity with this disorder. While 62.4% of the GPs rated their familiarity with FH as above average and 91.4% reported being familiar with the FH treatment and referral guidelines, more than half of the GPs answered fewer than half of the 8 knowledge-related questions correct. The survey performance of the Dutch GPs was comparable to that of the UK primary care physicians with regard to their responses to the knowledge-related questions, although their self-rated familiarity with FH was higher than that in the other countries. Collectively, these findings underscore the need for further FH education and awareness programmes in primary care in the Netherlands in order to enhance FH detection and ensure appropriate treatment of FH patients.

### Limited knowledge constrains screening and identification of familial hypercholesterolaemia

The current study sheds light on the suboptimal knowledge of FH among Dutch GPs. These findings align with previous questionnaire studies conducted in the countries participating in the ‘Ten Countries Study’, as well as the US [13, 14, 18, 19]. A consistent pattern observed in these studies was that the primary care physicians perceive themselves as the most appropriate healthcare providers for managing FH, despite their suboptimal awareness of the guidelines and their limited knowledge of its prevalence and the diagnostic characteristics. Only a small proportion of GPs in the current study correctly identified the FH prevalence (19.5%) and heritability (55.7%), which are key factors for recognising potential FH cases. Most GPs either underestimated the true prevalence of FH, perceiving it to be less common than it actually is, or indicated uncertainty regarding the prevalence. In addition, only 13.6% correctly identified the CVD risk associated with FH, while the vast majority (53.4%) underestimated this risk. Moreover, GPs perceived the threshold for premature CVD onset to be 4 years earlier for women and 3 years earlier for men compared with the defined age thresholds outlined in the guidelines, although this difference in perception may not necessarily translate into a significant impact on clinical outcomes or management strategies. Nonetheless, the limited understanding of FH prevalence and heritability and the associated CVD risk poses a significant obstacle to effective FH screening and can further contribute to the problem of FH underdiagnosis and undertreatment [4]. These findings emphasise the necessity of increased awareness among GPs to ensure accurate recognition of FH cases.

### Clinical implications

Guidelines have recommended that early identification and management of FH patients take place in a primary care setting, as primary care plays a critical role in cascade screening and achievement of long-term treatment adherence [1, 20]. Indeed, most GPs acknowledged their suitability as healthcare provider to detect FH and were open to interventions that improve FH identification, including interpretative comments accompanying laboratory results to alert them to the likelihood of FH. Moreover, most GPs (~70%) expressed their intentions to screen lipid profiles in close relatives once an FH patient was identified. This holds substantial importance, as cascade screening has been established as a highly cost-effective mean of identifying new cases of FH [21]. Nonetheless, the findings revealed a disparity between the self-perceived familiarity and the actual knowledge of FH among Dutch GPs.

Therefore, it is imperative to develop strategies that address these knowledge gaps and enhance FH awareness among GPs. These might include a more pronounced integration of FH education into GP training curricula, dissemination of FH-related information through frequently attended GP conferences and webinars, and enhanced collaboration with lipid specialists. Focusing on GPs in the early stages of their career may especially be effective, considering that GPs with more professional experience achieved significantly higher knowledge scores, possibly due to exposure these more experienced GPs had to the nationwide cascade screening programme, which was discontinued in 2013. For GPs less experienced in FH management, more referrals to specialised lipid clinics and increased interaction with experts are essential until adequate knowledge is acquired.

Furthermore, the issue of suboptimal understanding of FH in primary care extends beyond the borders of the Netherlands and rather encompasses an international challenge. Therefore, exploring international collaboration to design effective educational programmes targeting knowledge barriers may offer a valuable approach to overcome global key barriers in FH care implementation. These educational programmes, preferably developed in collaboration with GPs, should be embedded in implementation science frameworks [22, 23]. Implementation science provides a structured method to integrate recommended strategies and guidelines into practice, focusing on identifying and addressing gaps between current practice and evidence-based recommendations. By leveraging these frameworks, which focus on factors that can create resistance to health system reform, such as resource constraints and computability with existing workflows, tailored interventions can be systematically designed, tested and implemented, which can ultimately improve the detection and management of FH [23].

### Study limitations

The current study has several limitations. First, the participants were opportunistically selected, potentially leading to a bias if GPs with a higher affinity towards the subject matter were more likely to respond. However, this would imply an overestimation of the participants’ performance, further emphasising the need for additional efforts to enhance FH knowledge and awareness among Dutch GPs. In addition, substantial efforts were undertaken to ensure the demographic representation of the sample concerning the GP population in the Netherlands.

Second, it is important to note that healthcare systems vary across countries. Particularly, the delivery of primary care may involve various types of physicians in different countries, whereas in the Netherlands, this role is exclusively fulfilled by GPs. Nevertheless, international guidelines assert that FH management should ideally be performed in a primary care setting.

## Conclusion

This study showed that, despite the relatively high FH detection rate in the Netherlands, there were important gaps in the knowledge of Dutch GPs regarding the prevalence and heritability of FH, the associated CVD risk and the FH treatment and referral guidelines. To enhance early detection of FH and ensure appropriate treatment of affected patients in primary care settings, it is crucial to develop effective educational strategies that can increase the FH knowledge and skills of GPs. Educational programmes should be developed in collaboration with GPs and imbedded in implementation frameworks.

### Supplementary Information

**Table S1** Questions included in the survey

**Figure S1** Distribution of familial hypercholesterolaemia knowledge scores among Dutch general practitioners
