# Supplementary material for: Unexpected gaps in knowledge of familial hypercholesterolaemia among Dutch general practitioners
Source: Neth Heart J. 2024 Apr 4;32(5):213–20. doi: 10.1007/s12471-024-01862-y (PMC11039606; doi:10.1007/s12471-024-01862-y)
Supplement: Supplementary file 1 — Table S1 Questions included in the survey [file 12471_2024_1862_MOESM1_ESM.docx]

**Table S1** Questions included in the survey

| **Awareness of Familial Hypercholesterolemia** |
| --- |
| 1. **On a scale of 1 to 7; how familiar are you with familial hypercholesterolemia?**  - 1 - 2 - 3 - 4 - 5 - 6 - 7 |
| 1. **Are you aware of guidelines on the detection and management of familial hypercholesterolemia? ***  - Yes - No - Partially |
| 1. **Are you aware of any specialist/clinical services for lipid disorders to whom you can**   **refer patients?**   - Yes - No |
| **Knowledge pertaining to Familial Hypercholesterolemia** |
| 1. **Which one description below best describes familial hypercholesterolemia?**  - The presence of family members with diagnosed high cholesterol - A genetic disorder that is characterized by very high cholesterol and a family history of premature heart disease - The presence of multiple lipid abnormalities that may be genetic in nature - An ultra­rare, potentially fatal condition caused by cholesterol levels that can be up to six times the normal level - Don’t know |
| 1. **Which one of the following lipid profiles is most consistent with the diagnosis of familial hypercholesterolemia? (Reference intervals: Total cholesterol <5.5mmol/L; Triglyceride <1.7mmol/L; HDL­cholesterol >1.0mmol/L; LDL­cholesterol <3.5mmol/L)**  - Total cholesterol 6.0mmol/L; Triglyceride 3.4mmol/L; HDL­cholesterol 0.8mmol/L; LDL­cholesterol 3.8mmol/L - Total cholesterol 6.3mmol/L; Triglyceride 12.2mmol/L; HDL­cholesterol 1.0mmol/L; LDL­cholesterol ­mmol/L - Total cholesterol 8.0mmol/L; Triglyceride 1.1mmol/L; HDL­cholesterol 1.0mmol/L; LDL­cholesterol 6.5mmol/L - Total cholesterol 5.4mmol/L; Triglyceride 1.3mmol/L; HDL­cholesterol 1.7mmol/L; LDL­cholesterol 3.1mmol/L - Total cholesterol 7.1mmol/L; Triglyceride 1.0mmol/L; HDL­cholesterol 3.5mmol/L; LDL­cholesterol 3.2mmol/L |
| 1. **What is the prevalence of familial hypercholesterolemia? ****  - 1 in 100 persons - 1 in 250 persons - 1 in 500 persons - 1 in 1,000 persons - 1 in 2,000 persons - 1 in 5,000 persons - Don’t know |
| 1. **What is the likelihood that first-degree relatives (i.e. parents, siblings and children) of**   **someone who has familial hypercholesterolemia will also have the condition**  **themselves?**   - 0% - 25% - 50% - 75% - 100% - Don’t know |
| 1. **How much greater is the risk of premature coronary heart disease (CHD) in untreated**   **familial hypercholesterolemia patients compared to the general population?**   - 2 times greater - 4 times greater - 10 times greater - 20 times greater - 50 times greater - Don’t know |
| 1. **When you are assessing a patient’s family history, at what age for men do you consider heart disease to be “premature”? Leave blank if you wish to answer "Don't know".**  - Premature heart disease in men (years of age or younger): |
| 1. **When you are assessing a patient’s family history, at what age for women do you consider heart disease to be “premature”? Leave blank if you wish to answer "Don't know".**  - Premature heart disease in women (years of age or younger): |
| 1. **Is the following statement true or false?** *“An accurate diagnosis of familial hypercholesterolemia can only be made via genetic test.”*  - True - False - Don’t know |
| **Practice, Preference, and Screening Familial Hypercholesterolemia ***** |
| 1. **In your view, which healthcare providers would be most effective at early detection of familial hypercholesterolemia and screening first-degree relatives? (Please tick up to two)**  - Lipid specialists - General practitioner - Cardiologists - Nurses with experience in cardiac risk prevention - Pediatricians - Obstetricians/Gynecologists - Endocrinologists - Other: |
| 1. **In patients with documented premature coronary artery disease which of the**   **following do you routinely carry out? (Please tick all that apply)**   - Look for arcus cornealis - Look for tendon xanthomata - Take a detailed family history of coronary artery disease - Screen close relatives for hypercholesterolemia - All of the above - None of the above |
| 1. **If you have patients with familial hypercholesterolemia under your care do you**   **routinely screen close relatives for this condition with a lipid profile?**   - Yes, patient’s children only - Yes, patient’s children and other close relatives - No - Not applicable |
| 1. **At what age would you test young individuals for hypercholesterolemia in a family**   **with premature coronary heart disease?**   - 0 – 6 years - 7 – 12 years - 13 – 18 years - None of the above - Don’t know |
| 1. **If yes to question 3, have you referred patients with familial hypercholesterolemia**   **to this service?**   - Yes - No - Don’t know |
| 1. **Which one of the following options could usefully assist you in detection of familial**   **hypercholesterolemia in your practice?**   - Laboratory report on a lipid profile alerting possible familial hypercholesterolemia - Alert by the clinical software system in your practice - Direct telephone call from the laboratory - All of the above - None of the above - Don’t know - Other (please specify): |
| **Demographics ****** |
| 1. **In which province is your practice located in the Netherlands?**  - Drenthe - Flevoland - Friesland - Gelderland - Groningen - Limburg - Noord-Brabant - Noord-Holland - Overijssel - Utrecht - Zeeland - Zuid-Holland |
| 1. **How many patients currently under your care, if any, have been formally diagnosed**   **with familial hypercholesterolemia?**   - Number of patients: |
| 1. **How would you describe the area of your practice?**  - Metropolitan - Rural |
| 1. **What type of practice do you work in?**  - Group practice - Duo practice - Solo practice |
| 1. **How many years have you been in practice since completing your medical degree?**  - Number of years: |
| 1. **Approximately how many patients do you see for any condition in an average**   **month?**   - Number of patients: |
| 1. **How many of your patients (do you estimate) have been formally diagnosed with familial hypercholesterolemia?**  - Number of patients: |

Questionnaire was adapted from the survey employed by Pang et al. in the ‘Ten Countries Study’ [10]. *This question was modified as follows: an additional response option of ‘Partially’ was included. **This question was modified as follows: an additional response option of ‘1:250’ was included. ***Questions on treatment were excluded in this section on practices care for familial hypercholesterolemia. ****The questions on demographics were tailored to the Netherlands. HDL, high-density lipoprotein; LDL, low-density lipoprotein
