# Supplementary material for: Unexpected gaps in knowledge of familial hypercholesterolaemia among Dutch general practitioners
Source: Neth Heart J. 2024 Apr 4;32(5):213–20. doi: 10.1007/s12471-024-01862-y (PMC11039606; doi:10.1007/s12471-024-01862-y)
Supplement: Supplementary file 2 — Figure S1 Distribution of familial hypercholesterolaemia knowledge scores among Dutch general practitioners [file 12471_2024_1862_MOESM2_ESM.docx]

**Figure S1** Distribution of familial hypercholesterolaemia knowledge scores among Dutch general practitioners


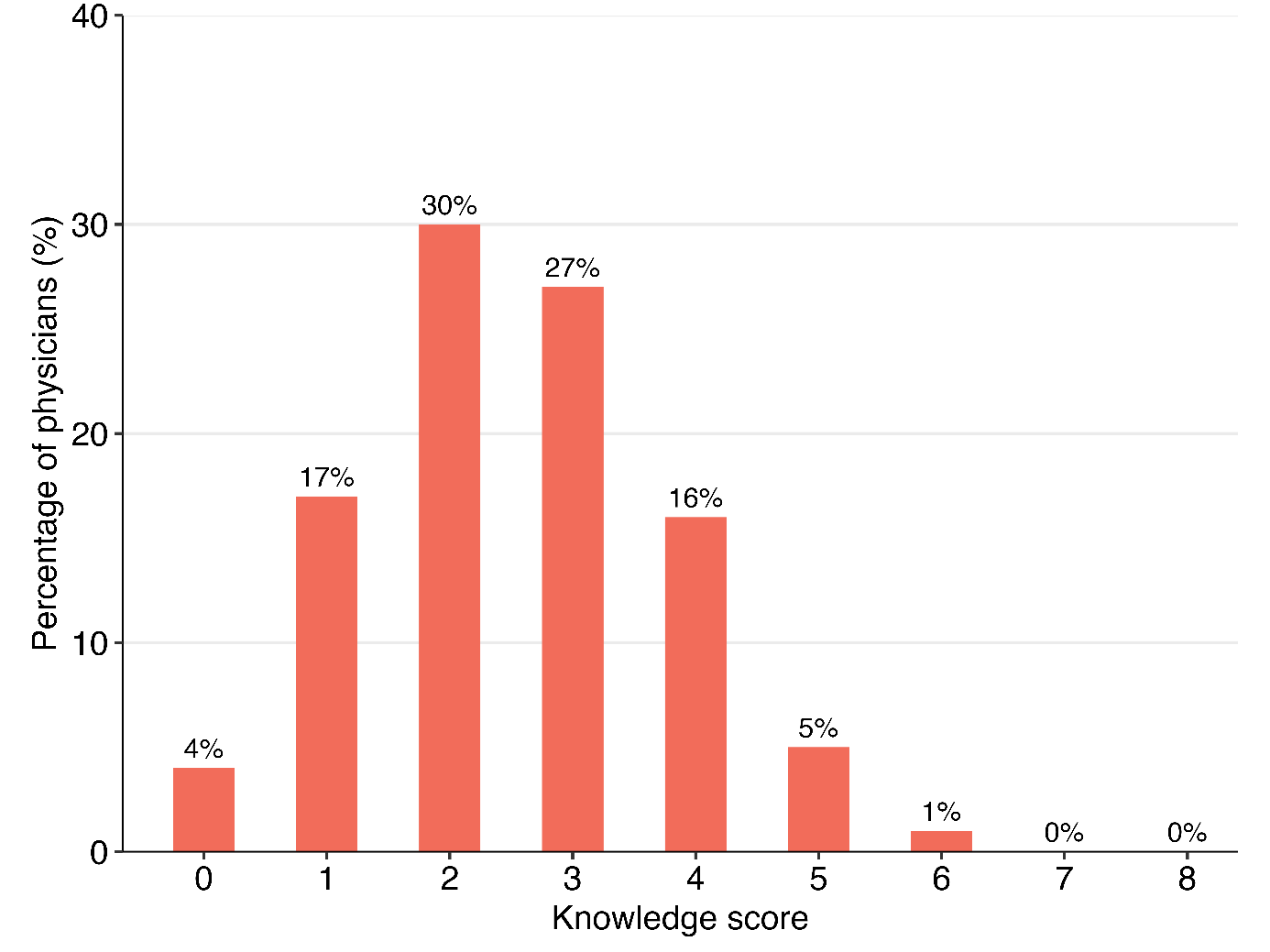


The figure depicts the distribution of knowledge scores among general practitioners, expressed as a percentage.
